# Recovery of Ga(III) by Raw and Alkali Treated *Citrus limetta* Peels

**DOI:** 10.1155/2014/968402

**Published:** 2014-07-24

**Authors:** Sachin C. Gondhalekar, Sanjeev R. Shukla

**Affiliations:** Department of Fibres and Textile Processing Technology, Institute of Chemical Technology, Nathalal Parekh Marg, Matunga, Mumbai 400019, India

## Abstract

Alkali treated *Citrus limetta* peels were used for recovery of Ga(III) from its aqueous solution. The raw and alkali treated peels were characterized for functional groups. The efficiency of adsorption increased from 47.62 mg/g for raw peels to 83.33 mg/g for alkali treated peels. Between pH 1 and 3, the adsorption increased and thereafter decreased drastically. The adsorption followed pseudosecond order kinetics and Langmuir isotherm gave the best fit for the experimental data. Desorption studies showed 95.28% desorption after 3 cycles for raw peels while it was 89.51% for alkali treated peels. Simulated Bayer liquor showed 39.57% adsorption for gallium ions on raw peels which was enhanced to 41.13% for alkali treated peels.

## 1. Introduction

Gallium is the 30th in terms of abundance at an average concentration of 19 mg/g. It finds significant applications in the semiconductor industry through use of gallium arsenide and gallium nitride. Gallium is classified as a “strategic” metal, since it is used in high-tech gadgets like microwave transceivers, DVDs, laser diodes in CDs, and so forth and defence-related activities [1–3]. Gallium nitrate and gallium citrate are used in medical imaging as radio contrast agents [4]. Gallium arsenide is used to make rectifiers and amplifiers. Due to good adhesion to glass and high reflectivity, gallium is used in high quality mirrors [5]. There are no gallium-containing minerals of any economic significance. Due to its uniform distribution in soil, its extraction is uneconomical. It is usually associated with aluminium in bauxite, nephelines, and other ores and recovered as a by-product while producing alumina. The United States Geological Survey has estimated total world's primary gallium production to be about 273 tonnes in 2012 [6], the estimated consumption being 280 tonnes. As per the report of Roskill Information Services, neomaterial estimated that 50% of gallium consumed worldwide in 2010 came from recycled sources [7]. As per working Group Report of 12th Five Year Plan, India produced around 55 kg of gallium in recent years. Two plants, namely, Hindalco Industries Ltd. at Renukoot and National Aluminium Co. Ltd., at Damanjodi Alumina Refinery, Odisha, recover gallium [8]. It is derived from wastes of industrial processes, such as flue dusts from the zinc industry, waste generated during smelting of phosphate to produce elemental phosphorus, or sludge from the aluminium industry. Since aluminium lies in the same group of periodic table, gallium has affinity towards it. Bauxite (the primary aluminium ore) typically contains 0.003 to 0.01% gallium. Concentrations in zinc ores (e.g., sphalerite) are comparable. Since primary sources are scarce, the general strategy is to recover gallium from intermediate industrial products.

Bayer liquor is one of the important resource for recovery of gallium. About 90% of the world's primary gallium is produced from Bayer liquor [9]. In the Bayer process, about 70% of the gallium content of bauxite is leached along with aluminium and about 30% is retained in the red mud. Gallium accumulates in the Bayer liquor in successive cycles, attaining concentrations of 100–200 mg/L [10]. Gallium is also obtained from the iron mud or residues that result from the purification of zinc sulphate solutions, in zinc production. There are four kinds of recovery methods for gallium, namely, fractional precipitation, electrochemical deposition, solvent extraction, and ion exchange. Fractional precipitation is a complex process; solvent extraction is efficient but very slow; electrochemical method uses mercury cathode which is banned due to mercury toxicity, whereas ion exchange gives effective recovery of gallium. Industrial resins such as Duolite ES-346 and PHG586 may be used; however, the process is very expensive. Hence, further research for cheap, efficient, and environmentally friendly recovery of gallium is essential [11].

Biosorption studies have mainly focused on the removal and recovery of heavy metal ions from industrial effluents, detoxification being their primary goal. Functional groups present in the biosorbents that are responsible for metal binding are carboxyl, phosphoryl, sulfhydryl, amino, sulfate, imidazole, thioether, phenol, amide, and hydroxyl in various biomolecules of peptide, protein, and polysaccharide moieties of the cell walls. These functional groups bind the metal ions mainly by adsorption, ion exchange, and chelating effects [12].

Very few reports have been published on the removal/recovery of gallium using natural adsorbent [13–15].* Citrus limetta* peels are a natural pectinacious agrowaste material, typically generated in large quantities by the fruit juice industry, rich in pectin with abundant presence of carboxyl groups. It is reported that these peels have a metal binding mechanism similar to brown algal biosorbents since pectin is chemically similar to the brown algal cell wall polysaccharide alginate [6, 7]. These peels have been found to be very good adsorbents for Pb(II) and Cd(II) [16–18]. Our previous studies have shown good binding capacity by citrus peels for Pb(II) ions [18].

In the present work, the adsorption of Ga(III) from the aqueous solution of gallium nitrate and from the simulated Bayer liquor on* Citrus limetta* peels, in its raw as well as alkali treated form, has been reported.

## 2. Materials and Methods

### 2.1. Materials

*Citrus limetta* fruit peels procured from local juice shop were used as biosorbents. Demineralized water was used throughout the experiment for dilution and washing purposes. Stock solution of 1000 mg/L was prepared from gallium nitrate salt (Sigma-Aldrich, India). Gallium standard used for calibration of Atomic Absorption Spectrometer (Model: GBC 932plus, Austria) was supplied by Merck Inc., Germany.

pH adjustment of metal ion solution was achieved by appropriate addition of 0.1 M NaOH and 0.1 M HCl.

### 2.2. Methods

#### 2.2.1. Preparation of Biosorbent

*Citrus limetta* peels procured from local juice shop were soaked in water for 4 h, washed, and cleaned using 1% nonionic detergent solution and again with demineralised water. The washed peels were dried at 60°C till constant weight, reduced to small particle size using a grinder, sieved to mesh sizes between 425 and 800 *μ*, and stored in zip lock bags. The biomass thus obtained was termed as raw citrus peels (RCP).

#### 2.2.2. Modification of Functional Groups on RCP

The raw peels were treated with different chemical agents as reported to enhance their adsorption capacity. This data is further presented in Table 1 [19].

RCP was treated with 0.05 M aqueous NaOH for 3 h at room temperature. It was then washed thoroughly with demineralized water till neutral pH and then dried in an oven at 60°C for 24 h and stored in zip lock bags to avoid contamination with moisture. It was termed as alkali treated citrus peels (ACP). The weight loss of biomass due to alkali treatment was estimated experimentally.

#### 2.2.3. Estimation of Gallium Ions

Ga(III) concentration in aqueous solution was determined by Atomic Absorption Spectrometer (GBC 932 plus, Australia) at 287.1 nm with an air-acetylene flame. Each time, AAS was calibrated by using standard 1000 mg/L Ga(III) solution.

#### 2.2.4. ATR-IR Spectroscopy

The functional groups present on the surface of RCP and ACP were determined by recording the infrared spectra of these peels using Shimadzu 8400S FT-IR spectrometer (Figure 2).

#### 2.2.5. Scanning Electron Microscopy (SEM)

Microscopic images of RCP and ACP were taken by scanning electron microscope (Jeol JSM 6380 LA spectrometer, Tokyo, Japan) to reveal the morphological aspects of the surface of the biomass samples.

#### 2.2.6. Estimation of Acidic Groups

Number of acidic sites present on RCP and ACP were estimated by methylene blue absorption method [20, 21]. When a biomass is treated with a cationic dye like methylene blue, the coloured cations of the dye are quantitatively absorbed by acidic groups present on the material forming strong ionic linkage. A weighed sample of the biomass was added to an Erlenmeyer flask containing 25 mL of aqueous methylene blue chloride solution (300 mg/L) and 25 mL borate buffer of pH 8.5. It was kept for 3 h at 25°C and then 5 mL of filtered sample was transferred to a volumetric flask containing 10 mL 0.1 M HCl followed by dilution to 100 mL. The decrease in the intensity of colour was measured on UV-visible spectrometer (Techcomp; UV-VIS 8500) at the *λ*_max⁡_ = 650 nm. Using the calibration plot, the amount of unabsorbed methylene blue was calculated. The value gives quantitative information about the acidic sites present on the surface of peels.

#### 2.2.7. Batch Wise Adsorption Experiments

Unless otherwise stated, for all the batch wise adsorption experiments, 100 mg of dry adsorbent was placed in 40 mL Ga(III) solution having varying initial concentrations ranging from 40 mg/L to 200 mg/L at pH 3.0 in a 250 mL stoppered Erlenmeyer flask and agitated in an orbital shaker (Rossari Biotech Ltd., Mumbai) at 150 rpm for 4 h at room temperature. The biomass was then allowed to settle down and the supernatant solution was pipetted out. The solutions were estimated for the remaining Ga(III) concentration using flame type AAS.

The biosorption capacity of gallium per unit of dry biomass (mg of Ga/g of dry biomass) was calculated by using

(1) $$\begin{array}{c} q_{\text{eq}}=\frac{\left(C_{0}-C_{\text{eq}}\right)V}{W}, \end{array}$$

where *q*_eq_ is the equilibrium adsorption capacity (mg/g), *C*_0_ and *C*_eq_ are the initial and equilibrium concentrations of Ga(III) (mg/L), respectively, *V* is the volume of adsorbate (L), and *W* is the mass of adsorbent (g).

The biosorption efficiency, *E*%, of the metal ion was calculated from

(2) $$\begin{array}{c} E\%=\frac{\left(C_{0}-C_{\text{eq}}\right)}{C_{0}}\times 100. \end{array}$$

The optimum pH for the biosorption was evaluated by adjusting the pH between 1 and 3 with an interval of 0.5 using 1 M HCl.

The kinetics of adsorption of Ga(III) was studied by shaking 2.5 g of adsorbent samples with 200 mL of approximately 100 mg/L solutions of Ga(III) at pH 3.0 and at room temperature (30°C) up to 300 min in a 250 mL Erlenmeyer flask at 150 rpm. At each predetermined time, 1 mL of the solution was removed and tested for its metal content using flame type AAS. The amount of metal ions adsorbed was evaluated from the difference between the initial and final concentrations of Ga(III) ions in the solution.

#### 2.2.8. Isotherm Modeling

Out of many isotherm models, the Langmuir and Freundlich models are the most commonly studied due to their ease of interpretation [22]. Langmuir isotherm was initially derived for adsorption of gases on solid surfaces (Figure 6), and it considered sorption as a chemical phenomenon; the sorbent surface contains only one type of binding site and the sorption is limited to monolayer [23]. It is given by

(3) $$\begin{array}{c} q_{\text{eq}}=\frac{K_{l}q_{\max}C_{\text{eq}}}{1+K_{l}C_{\text{eq}}}, \end{array}$$

where *q*_eq_ is the metal uptake, *q*_max⁡_ is the maximum biosorption capacity, *K*_*l*_ is the constant related to adsorption energy, and *C*_eq_ is the equilibrium concentration of metal ions. The Langmuir parameters can be determined from the slope and the intercept of the plot *C*_eq_/*q*_eq_ versus *C*_eq_, based on the linearized form of the above equation, can be written as

(4) $$\begin{array}{c} \frac{C_{\text{eq}}}{q_{\text{eq}}}=\frac{1}{K_{l}q_{\max}}+\frac{C_{\text{eq}}}{q_{\max}}. \end{array}$$

Freundlich proposed an empirical isotherm relation as expressed in

(5) $$\begin{array}{c} q_{\text{eq}}=K_{f}C_{\text{eq}}^{1/n}, \end{array}$$

in which *K*_*f*_ and *n* are Freundlich constants (Figure 7). As the Freundlich isotherm is exponential, it can be reasonably applied only in the low to intermediate concentration range [24]. The equation can be linearized as

(6) $$\begin{array}{c} \log q_{\text{eq}}=\log K_{f}+\frac{1}{n}\log C_{\text{eq}}. \end{array}$$

Besides these two common adsorption isotherms, Sips isotherm is also widely used to study the biosorption mechanism of pectin containing compounds [25] (Figure 8). This isotherm is a combination of Langmuir and Freundlich isotherm equations, deduced for predicting the heterogeneous adsorption systems and to overcome the limitation of the rising adsorbate concentration associated with Freundlich isotherm model. At low adsorbate concentrations, it reduces to Freundlich isotherm; while, at high concentrations, it predicts a monolayer adsorption capacity characteristic of the Langmuir isotherm (Tables 4 and 5). As a general rule, the equation parameters are governed mainly by the operating conditions such as the alteration of pH, temperature, and concentration. Sips isotherm can be expressed as

(7) $$\begin{array}{c} q_{\text{eq}}=q_{\max}\frac{K_{s}C_{\text{eq}}^{n_{s}}}{1+K_{s}C_{\text{eq}}^{n_{s}}}, \end{array}$$

in which *n*_*s*_ is the Sips constant [26] (Table 6).

#### 2.2.9. Kinetic Modeling

Biosorption data under nonequilibrium conditions is usually described by pseudofirst order and pseudosecond order equations [27].

*Pseudofirst Order Equation*. The pseudofirst order kinetic equation or Lagergren equation is given as

(8) $$\begin{array}{c} \frac{dq_{t}}{dt}=k_{1}\left(q_{\text{eq}}-q_{t}\right), \end{array}$$

in which *q*_*t*_ is the amount of adsorbate adsorbed at time *t*, *q*_eq_ is the value at equilibrium, and *k*_1_ is the constant [28].

*Pseudosecond Order Equation*. The pseudosecond order kinetic equation has been frequently employed to analyze the biosorption data using different adsorbates and biosorbents as reviewed by Ho and McKay [29]. Consider

(9) $$\begin{array}{c} \frac{dq_{t}}{dt}=k_{2}{\left(q_{\text{eq}}-q_{t}\right)}^{2}, \end{array}$$

in which *k*_2_ is a constant.

#### 2.2.10. Adsorption-Desorption Cycles

Repetitive adsorption-desorption studies were carried out to evaluate the economic feasibility of the process. Desorption of Ga(III) from previously adsorbed peels was carried out by shaking them with 40 mL of desorbing media at 30°C for 240 min. Different desorbing media with varied concentrations, studied for the desorption, were HCl, HNO_3_, and H_2_SO_4_. The metal ion content in the desorbing media was then estimated using AAS.

#### 2.2.11. Regeneration of Biosorbent

RCP and ACP were thoroughly washed with demineralized water after desorption, treated with 40 mL of 0.025 M NaOH solution for 240 min, washed again, dried at 60°C in hot air oven for 24 h, and reused for another adsorption-desorption cycle.

#### 2.2.12. Preparation of Simulated Bayer Liquor

The process feasibility was checked by adsorbing the Ga(III) ions from the simulated spent Bayer liquor on RCP and ACP. It was prepared in laboratory by adding the components which are generally present in spent Bayer liquor in their stoichiometric amount [30]. Thus, 25 g of Na_2_CO_3_ were dissolved in hot water and cooled to room temperature. To this, 125 g of NaOH pellets were added slowly with stirring followed by addition of 120 g of Al(OH)_3_. The solution was heated near to boiling point, cooled, filtered, and diluted to 1 L. Then 14.7 mL of gallium stock solution (13.62 g/L) was transferred into the solution and the solution was made up to 1 L by adding water. Forty mL of this simulated spent Bayer liquor was taken in an Erlenmeyer flask, with pH adjusted to 3.0 by 0.1 M HCl, and subjected to adsorption on 0.1 g of biomass. The concentration of Ga(III) ions before and after the adsorption was estimated on AAS. Weight loss of the biomaterials during adsorption process was also estimated gravimetrically.

## 3. Results and Discussion

### 3.1. Effect of Pretreatment on Ga(III) Biosorption

Citrus peels are mainly made up of pectin (around 30% by mass), which is rich in galacturonic acid [31]. In nature, around 80% of carboxyl groups of galacturonic acid are present in esterified form as methyl carboxylate. The nonesterified galacturonic acid units can be either free acids (carboxyl groups) or salts with sodium, potassium, or calcium. The salts of partially esterified pectins are called pectinates. Salts below 5% degree of esterification are called pectates and the insoluble acid form is the pectic acid [32]. Alkali treatment is very effective in hydrolysis of these esterified galacturonic acid units and converts them into free acid sites. It also ruptures the cell wall and exposes more functional groups, thereby promoting the heavy metal adsorption by preferential ion-exchange mechanism [33]. Earlier oxidative pretreatment of cellulosic biomass such as jute and coir with hydrogen peroxide has shown to enhance the adsorption capacity for Pb(II) cations [34, 35]. Oxidized coir also showed enhanced adsorption of gallium (19.42 mg/g) compared to unmodified coir (13.75 mg/g) [13].

Raw* Citrus limetta* peels (RCP), a pectin containing waste biomaterial, have shown good potential to adsorb Pb(II) ions [18]. Initial experiments on RCP also showed the potential to adsorb Ga(III) ions from its aqueous solution (*C*_eq_ = 21.70 mg/g for *C*_*i*_ = 70 mg/L). RCP was given various chemical treatments that are reported to enhance the adsorption capacity of biomaterials [19] (Table 1).

**Figure 1:**
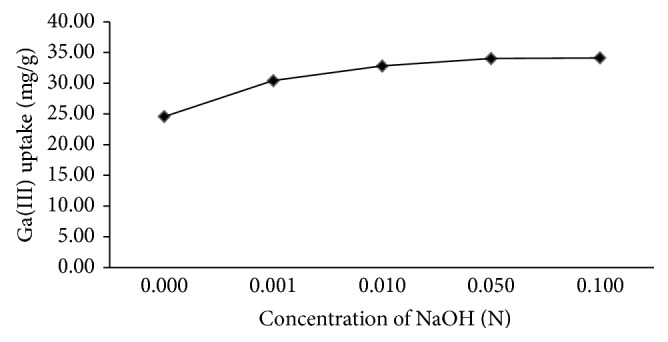
Effect of different concentration of NaOH on biosorption capacity of* Citrus limetta* peels (*C*_*i*_ = 100 mg/L, *t* = 4 h, *T* = 30°C).

**Figure 2:**
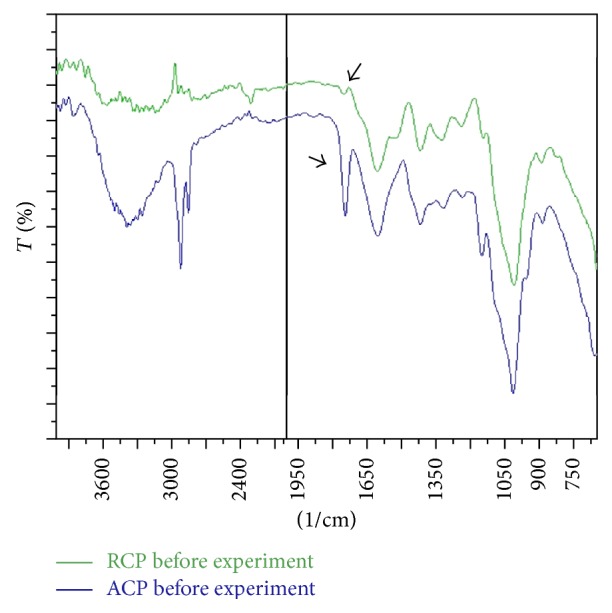
IR plot of RCP and ACP.

Among those, it was found that the alkali treatment was the most efficient in enhancing the adsorption capacity of peels and hence the concentration of NaOH was optimized at RT (30°C) and 2 h treatment time (Figure 1) (Table 2). Different acidic groups such as carboxylic and sulphonic acid also get converted into their sodium forms which have been shown to promote heavy-metal ion adsorption [36].

### 3.2. FT-IR Spectra of Biomass

FTIR spectra of peels showed peak at ~3350 cm^−1^ which is characteristic of hydroxyl group, mainly due to water. The peak observed at ~1625 cm^−1^ is attributed to asymmetric stretching of the carboxylic (C=O) double bond. The peaks observed at 2924 cm^−1^ and 2856 cm^−1^ in ACP are due to asymmetric and symmetric stretching modes of methylene groups. No strong shift in the wave number ~1750 cm^−1^ was observed in RCP, although the intensity of peak increased in ACP which is characteristic of carbonyl group of carboxylic acid (–COOH) and ester (–COOR). This indicates increase in the number of carboxylic groups in ACP which has also been quantified by methylene blue absorption method to 6.32 × 10^−2 ^mmol/g from 5.40 × 10^−2 ^mmol/g in RCP. Thus, the alkali treatment caused modification of the ester functional groups to carboxylic acid groups present in raw peels.

### 3.3. Scanning Electron Microscopy

**Figure 3:**
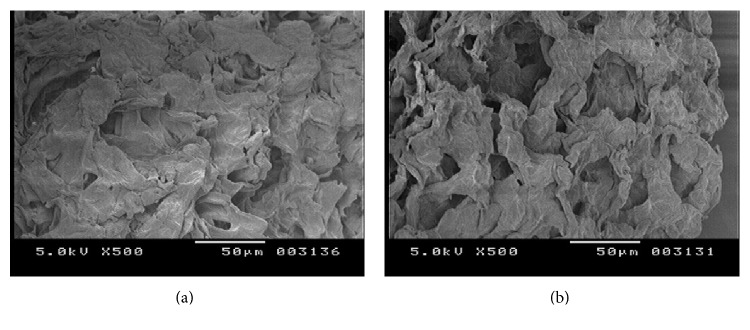
Scanning electronic micrographs of (a) RCP and (b) ACP with magnification 500x.

SEM images clearly indicate the morphological changes in the peels on alkali treatment. It can be easily depicted from the SEM that the surface of peels is highly heterogeneous. As seen in Figure 3, ACP surface shows more cavities and more number of opened up pores as compared to RCP indicating that it has increased the surface area available for adsorption. Alkali treatment proved to be effective in rupturing the cell walls due to which more functional groups are exposed on the surface and became available for adsorption. Apart from that, no further significant morphological changes were apparent in the SEM images.

### 3.4. Estimation of Acidic Groups on the Surface of Peels

**Figure 4:**
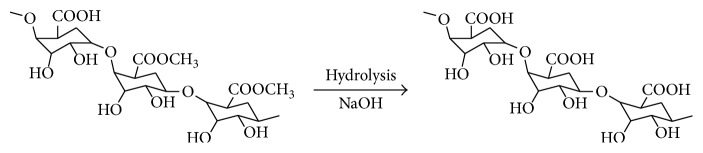
Structure of pectin before and after alkaline treatment.

The carboxyl groups present in the biosorbents have been proved to be directly responsible for the sorption of heavy metals [17, 18]. These are the most abundant acidic functional groups and the adsorption capacity of peels is directly related to the presence of these sites in pectin in the form of galacturonic acid. Estimation of acidic groups on the surface of peels available for sorption reveals that ACP contains contains more carboxylic acid groups (6.32 × 10^−2 ^mmol/g) as compared to RCP (5.40 × 10^−2 ^mmol/g). This has been attributed to the alkaline hydrolysis of some of the ester groups present on the surface of the peels under the treatment conditions. Structure of pectin before and after hydrolysis is as shown in Figure 4.

### 3.5. Effect of Initial Solution pH

pH is one of the most crucial factors which drives the efficiency of adsorption, as it governs the speciation of the metal ions in aqueous solution and also determines the degree of protonation on the biomass. At lower pH, protons compete with the metal ions thereby decreasing the adsorption, while, at higher pH, metal ions form corresponding hydroxides get precipitated out.

**Figure 5:**
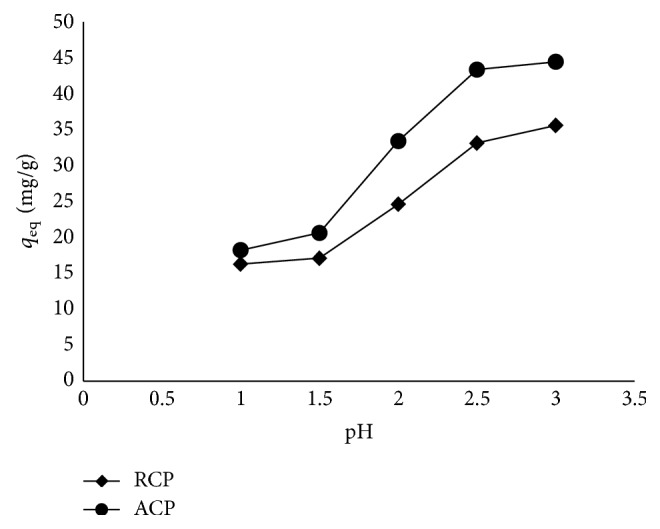
Effect of pH on biosorption of Ga(III).

**Figure 6:**
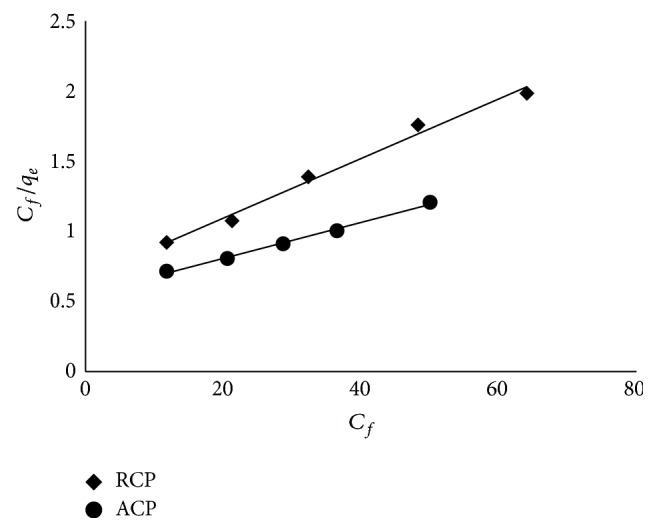
Linearized Langmuir isotherm.

**Figure 7:**
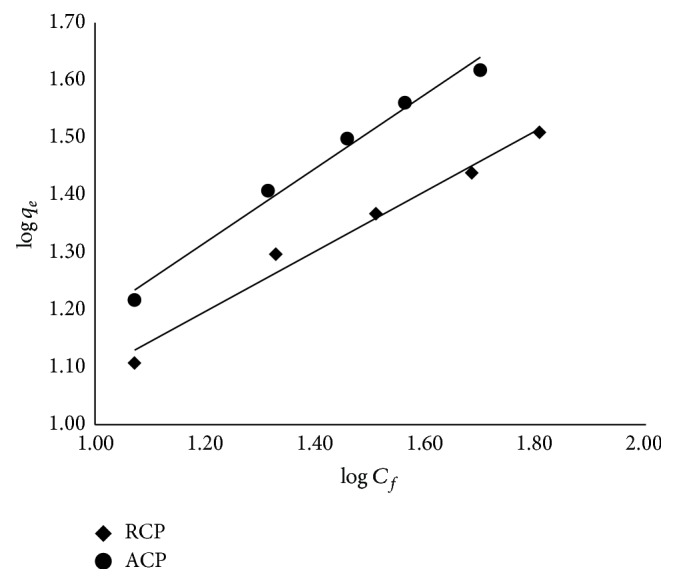
Linearized Freundlich isotherm.

**Figure 8:**
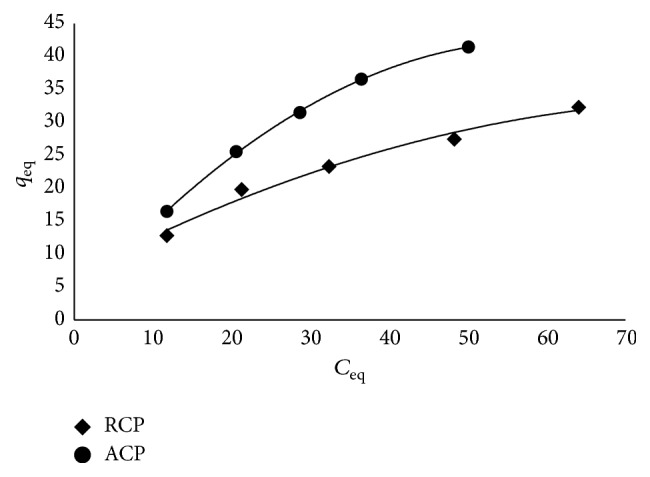
Nonlinear Sips isotherm.

The biosorption of Ga(III) was strongly affected by initial solution pH. The sorption capacity of RCP has increased from 16.29 mg/g at pH 1.0 to 35.59 mg/g Ga(III) at pH 3.0 (Figure 5) indicating an increase in adsorption capacity. Beyond pH 3.0, precipitation occurs, as gallium forms a hydroxide gel and loses its solution characteristics [36]. In case of ACP, the increase in adsorption capacity registered was from 18.23 mg/g to 44.44 mg/g at similar pH values.

In aqueous solution, gallium is always present in its hydrated form with six molecules of water held strongly to make an octahedral complex. The strength of metal-oxygen bond weakens O–H bond; hence, hydrolysis occurs and protons are released, thus giving acidic solution. The more metal ion concentration in the solution is, the more the hydrolysis is and, hence, the more acidic the solution becomes [37, 38]. Ga(III) ions are Lewis acids, and, in aqueous solution, they form aqua ions of the formula Ga(H_2_O)_6_^3+^. The aqua ions undergo hydrolysis; the hydrated gallium ions have six molecules of water which are held firmly giving an octahedral complex. The first hydrolysis step is given generically as

(10) Ga(H2O)63++H2O⇌[Ga(H2O)5(OH)]+2+H3O+.

Thus, the aqua cations behave as acids in terms of Brønsted-Lowry acid-base theory. This effect is easily explained by considering the inductive effect of the positively charged metal ion, which weakens the O–H bond of an attached water molecule, causing the liberation of a proton relatively easy to make the solution acidic.

Like other metal ions, the adsorption of Ga(III) is also highly influenced by pH [39–41]. At lower pH, the affinity towards the proton of the binding site of peels is much greater than that of the metal ion (H^+^ ≫ M^3+^), compared with that at higher pH where M^3+^ ≫ H^+^ [36]. Therefore, the adsorption is less at a lower pH of 1.0 which remains almost unaffected till pH 1.5 as Ga(III) has strong competition with protons and then it increases with the pH as the concentration of protons decreases till pH 2.5 is attained.

From the data given in Table 3, it is clear that the adsorption of Ga(III) on* Citrus limetta* peels is much higher than any other biosorbent used in earlier studies [13]. This may be attributed to enhanced carboxylic acid sites in ACP pectin.

### 3.6. Adsorption Isotherm

The adsorption data were fitted to Langmuir and Freundlich adsorption isotherms. The metal loading capacities (*q*_max⁡_) of RCP and ACP as calculated from the slope of the plot *C*_eq_/*q*_eq_ versus *C*_eq_ were found to be 47.62 and 83.33 mg/g, respectively. Though the data seems to be fitting equally well with the Langmuir (*r* ^2^ = 0.99) and Freundlich (*r* ^2^ = 0.99) isotherms, RMSE error value for Langmuir plot is much lower than Freundlich plot. This suggests that the Langmuir model gives better fit for both the adsorbents. “*K*_*f*_” and “*n*” were calculated from the intercept and slope of the plot log *q*_eq_ versus log *C*_eq_. Freundlich isotherm values of “*n*” were 1.61 and 1.90 for RCP and ACP, respectively, suggesting favourable adsorption by ACP.

### 3.7. Kinetic Modelling

**Figure 9:**
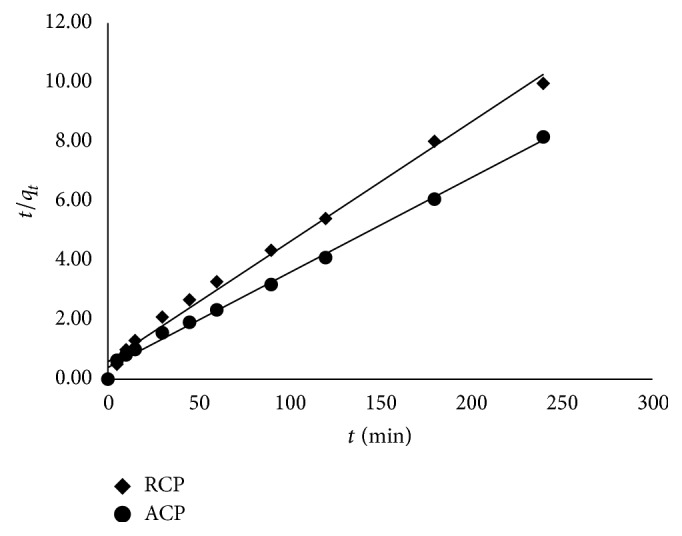
Pseudosecond order plot for *C*_0_ = 100 mg/L (RCP) and *C*_0_ = 100 mg/L (ACP).

Pseudosecond order (*R* ^2^ = 0.99) plot shows better fit than pseudofirst order (*R* ^2^ = 0.94) for RCP and ACP.  Figure 9 represent the linearized plots for second order model for RCP and ACP. Higher value of initial rate, *h*, in ACP (2.50 mg/g·min) suggests favorable adsorption. The values for pseudosecond order model has been listed in Table 7. The kinetic study reveals closeness of the predicted and experimental *q*_max⁡_ values, which are 25.00 and 24.11 for RCP and 32.26 and 29.77 for ACP, respectively. Pseudosecond order plot gives best fit for RCP as well as ACP; hence, it was considered that adsorption of Ga(III) on RCP and ACP takes place via pseudosecond order kinetics.

### 3.8. Desorption Studies

Desorption efficiency of the adsorbent is crucial to the recovery of gallium. The metal ion loaded adsorbent was most effectively desorbed by using 0.5 M HCl, when different acids (HCl, H_2_SO_4_, and HNO_3_) with different concentrations (0.1 M–1.0 M) were tried. Ga(III), adsorbed on the surface of the adsorbent, exchanges with the H^+^ ion of the acid due to its high affinity towards the functional groups, behaving as a cation exchanger. Desorption in the first cycle was observed to be more than 95%, which decreased marginally in the subsequent cycles. The weight loss study of the adsorbents during the adsorption-desorption cycles indicated that, after the first complete cycle, the weight loss for RCP was 19.19% while that for ACP was 17.53%. It increased to 37.05% for RCP and to 55.45% for ACP after the second cycle. Even after the third cycle, the same adsorbent can be used by seeing its good adsorption capacity.

### 3.9. Regeneration Studies

Although the* Citrus limetta* peels is a waste material obtained from the orange fruit, any chemical treatment to convert it into a better adsorbent adds to the cost. In order to make the process economically viable, the peels after desorption of Ga(III) were regenerated using 0.025N NaOH and reused for another adsorption-regeneration cycle. Low concentration of alkali is used as regeneration media for economic viability. Regeneration of the peels was studied for 3 cycles and showed comparative adsorption in the next cycles.

### 3.10. Simulated Bayer Liquor Sample Analysis

Synthetic Bayer liquor sample was analyzed for the efficiency of biosorbent for adsorption-desorption cycles (Table 9). It was observed that RCP showed 39.57% adsorption of gallium from the simulated spent Bayer liquor while ACP showed 41.13% adsorption. Both the adsorbents showed nearly complete desorption in 0.5 M HCl as desorbing media. The weight loss of biomass after first cycle was observed to be 29.98% for RCP and 18.03% for ACP. The decrease in the adsorption capacity as compared to pure aqueous solution of gallium may be attributed to the presence of other metal ions such as Na(I) and Al(III) present in the solution. These results are presented in Table 8. It may be seen that RCP and ACP are competitive to synthetic ion exchange resin showing adsorption capacities of 7.32 and 7.61 mg/g, respectively, which are higher than that of reported value (6.0 mg/g) on resin with hydroxamic acid ligand [3].

## 4. Conclusion

Waste biomass* Citrus limetta* peels were effectively used to adsorb gallium ions from aqueous solution. When treated with sodium hydroxide, the alkali treated peels (ACP) showed enhanced metal removal capacity. Optimum pH for adsorption was found to be 3.0. Kinetic data showed that the equilibrium reached within 180 minutes. Pseudosecond order model proved to be the best fit whereas isotherm studies reveal that the metal ion adsorption capacity of 47.62 mg/g for RCP was enhanced to 83.33 mg/g for ACP. The adsorbents showed good stability up to three adsorption-desorption cycles and hence proved practical to use at scale up level. Also RCP and ACP showed significant adsorption in synthetic Bayer liquor sample which highlights the use of* Citrus limetta* peels at commercial level.

## Figures and Tables

**Table 1:** Adsorption capacities of *Citrus limetta* peels (*C*_*i*_ = 100 mg/L, *t* = 4 h, and *T* = 30°C) on chemical modification.

Modification	Metal uptake (mg/L)
Nil (RCP)	24.40
1 M malonic acid treated peels	24.31
1 M oxalic acid treated peels	21.67
20% H_2_O_2_ treated peels	30.78
0.1 M sodium hydroxide treated peels (ACP)	34.59
1 M citric acid treated peels	23.90
1 M succinic acid treated peels	27.05
Acetylated peels	27.08
Chlorosulphonic acid treated peels	23.00
Methane sulphonic acid treated peels	23.33
0.1 M potassium dichromate treated peels	27.12

**Table 2:** Effect of concentration of NaOH on weight loss of biosorbent.

NaOH concentration (N)	Weight loss (%)
0.000	00.0
0.001	19.0
0.010	19.2
0.050	19.6
0.100	20.8

**Table 3:** Effect of biosorbent on adsorption of Ga(III) from aqueous solution.

Biosorbent used	Initial Ga concentration (mg/L)	*q*_max⁡_ (mg/g)	Reference
Oxidized coir	203	19.42	[13]
Alkali treated peels	200	76.26	This study

**Table 4:** Values obtained from Langmuir and Freundlich isotherms (linear parameters).

Adsorbent	Langmuir isotherm				Freundlich isotherm			
	*q*_max⁡_ (mg/g)	*b*	*r* ^2^	RMSE	*K*_*f*_	*n*	*r* ^2^	RMSE
RCP	47.62	0.031	0.99	3.02	3.84	1.61	0.99	7.08
ACP	83.33	0.022	0.99	3.69	3.69	1.90	0.98	7.72

**Table 5:** Values obtained from Langmuir and Freundlich isotherms (nonlinear parameters).

Adsorbent	Langmuir isotherm		Freundlich isotherms	
	*q*_max⁡_ (mg/g)	*b*	*K*_*f*_	*n*
RCP	46.54	0.033	4.04	0.50
ACP	76.26	0.024	4.11	0.60

**Table 6:** Values obtained from Sips isotherms.

Adsorbent	Sips isotherm			
	*K*_*s*_	*β*_*s*_	*a*_*s*_	*r* ^2^
RCP	1.124	1.212	0.018	0.99
ACP	2.412	0.787	0.038	0.99

**Table 7:** Experimental and pseudosecond order values for RCP and ACP.

Adsorbent	Experimental q _eq_ (mg/g)	*k*_2_ (×10^−3^/min)	q_eq_ (mg/g)	*r* ^2^	Initial rate, h (mg/g·min)
RCP	24.11	2.7	25.00	0.99	1.69
ACP	32.26	2.4	29.77	0.99	2.50

**Table 8:** Repeated adsorption-desorption cycles for Ga(III) adsorption.

Adsorbent	Cycle 1			Cycle 2				Cycle 3			
	% adsorption	% desorption	% weight loss	% adsorption	% desorption	% weight loss	Overall weight loss	% adsorption	% desorption	% weight loss	Overall weight loss
RCP	58.98	98.71	19.19	34.37	95.77	16.21	39.40	37.08	95.26	4.76	40.16
ACP	68.51	95.37	17.53	56.00	89.78	12.90	30.43	55.46	89.51	2.88	33.31

**Table 9:** Synthetic Bayer liquor adsorption-desorption cycle.

Adsorbent	Adsorption (%)	Desorption (%)	Weight loss (%)
RCP	39.57	98.35	29.98
ACP	41.13	92.23	18.03
